# Parental stress during pregnancy and maternity^*^

**DOI:** 10.1590/1980-220X-REEUSP-2022-0351en

**Published:** 2023-03-31

**Authors:** Carine Sanches Zani Ribeiro, Ellen Cristina Gondim, Luiz Guilherme Dacar Silva Scorzafave, Flávia Azevedo Gomes-Sponholz, Daniel Domingues dos Santos, Débora Falleiros de Mello

**Affiliations:** 1 Universidade São Paulo, Escola de Enfermagem de Ribeirão Preto, Programa de Pós-Graduação Enfermagem em Saúde Pública, Ribeirão Preto, SP, Brazil.; 2 Universidade São Paulo, Faculdade de Economia, Aministração e Contabilidade de Ribeirão Preto, Laboratório de Estudos e Pesquisas em Economia Social, Ribeirão Preto, SP, Brazil.

**Keywords:** Psychological Distress, Prenatal Care, Child Development, Primary Health Care, Distrés Psicológico, Atención Prenatal, Desarrollo Infantil, Atención Primaria de Salud, Angústia Psicológica, Cuidado Pré-natal, Desenvolvimento Infantil, Atenção Primária à Saúde

## Abstract

**Objective:**

To identify factors related to parental stress of women during pregnancy and the child’s first month of life.

**Method:**

Prospective longitudinal study in two stages. Analysis of home interviews with 121 participants, Gestational Stress Scale, and Parental Stress Scale. Fisher’s exact test, Spearman’s correlation, and linear and logistic multivariate regression were applied, with *p <* 0.05.

**Results:**

Most of the participants were between 18 and 35 years old, had 11 to 13 years of education, had no paid work, had a partner, usually the child’s father, planned pregnancy, were multiparous, and underwent prenatal care. During pregnancy, 67.8% had stress. In the first month after the child’s birth, most had low parental stress (52.1%). High parental stress correlated with some gestational stress. Planning pregnancy decreased parental stress.

**Conclusion:**

Gestational and parental stress in the child’s first month of life were correlated and pregnancy planning was a factor that reduced stress levels. Timely actions to reduce parental stress are essential for parenting and the child’s overall health.

## RESUMO

**Objetivo:**

Identificar fatores relacionados ao estresse parental de mulheres na gestação e primeiro mês de vida da criança.

**Método:**

Estudo longitudinal prospectivo em duas etapas. Análise de entrevistas em domicílio com 121 participantes, Escala de Estresse Gestacional e de Estresse Parental. Aplicados teste exato de Fisher, correlação de Spearman e regressão multivariada linear e logística, com *p <* 0,05.

**Resultados:**

A maior parcela das participantes tinha entre 18 e 35 anos, 11 a 13 anos de estudo, sem trabalho remunerado, com companheiro, geralmente o pai da criança, gestação planejada, multípara e realização do pré-natal. Na gestação, 67,8% apresentaram estresse. No primeiro mês após o nascimento do filho, a maioria teve baixo estresse parental (52,1%). Estresse parental alto apresentou correlação com algum estresse gestacional. Planejar a gestação diminuiu o estresse parental.

**Conclusão:**

Estresse gestacional e parental no primeiro mês de vida da criança foram correlacionados e o planejamento da gestação constituiu fator de diminuição dos níveis de estresse. Ações oportunas para reduzir o estresse parental são fundamentais para a parentalidade e saúde integral da criança.

## RESUMEN

**Objetivo:**

Identificar factores relacionados con el estrés parental de la mujer durante el embarazo y el primer mes de vida del niño.

**Método:**

Estudio longitudinal prospectivo en dos etapas. Análisis de entrevistas domiciliarias con 121 participantes, Escala de Estrés Gestacional y Estrés Parental. Se aplicó la prueba exacta de Fisher, correlación de Spearman y regresión multivariada lineal y logística, con *p <* 0,05.

**Resultados:**

La mayoría de las participantes tenía entre 18 y 35 años, tenía entre 11 y 13 años de escolaridad, no tenía trabajo remunerado, tenía pareja, generalmente el padre del niño, embarazo planificado, multípara y realizó control prenatal. Durante el embarazo, el 67,8% tuvo estrés. En el primer mes después del nacimiento del hijo, la mayoría presentó bajo estrés parental (52,1%). El estrés parental alto se correlacionó con algo de estrés gestacional. La planificación del embarazo disminuyó el estrés parental.

**Conclusión:**

El estrés gestacional y parental en el primer mes de vida del niño se correlacionaron y la planificación del embarazo fue un factor en la reducción de los niveles de estrés. Las acciones oportunas para reducir el estrés parental son esenciales para la responsabilidad parental y la salud general del niño.

## INTRODUCTION

Parental stress is indicated as a risk condition for child development and well-being, as well as for family dynamics^(1)^. This condition can affect parenting practices and the relationship between parental caregivers and children^(1,2)^. Promoting healthy child development, ensuring that all needs are met, and educating the child are challenges for families and daily situations, such as caring for children, marital relationships, financial demands, work, and the exercise of the roles of father and mother can generate stress^(3,4)^.

The term parental stress encompasses the imbalance of the parental role and may be present in the daily lives of mothers and fathers to some degree^(2)^. The stable and responsive parenting relationship in the first years of life protects the child from potential damage caused by excessive stress, generating safety for healthy development and an adult stage with skills to be resilient, deal with stress, and exercise self-regulation^(5)^. When adequate parenting is not achieved, the child may experience adversity and suffer losses in physical, emotional, educational, economic and social health^(1,3,5)^.

The period of very early childhood, from pregnancy to age three, is critical for the development of brain structures and circuits^(6)^. Stressful environments can interfere with the development process from the gestational stage, and it is important that the family context can provide opportunities for interventions to promote healthy development^(4,7)^.

The woman in the gestational period presents different feelings^(8)^ and, in situations of prenatal stress, changes may occur in the development of the fetus and, subsequently, of the child^(8,9)^. In addition, stress during pregnancy can affect the prenatal, postnatal bond, and parenting development^(5)^. Thus, the family environment requires attention to the experiences provided to children in this period^(10)^.

For professional practice in the field of Primary Health Care (PHC), elements that lead to parental stress and situations that reduce this stress shall be identified, to contribute to the expansion of good interactions between parental caregivers and children, reducing behavioral problems in childhood and improving the quality of family life and child development. These aspects assume great relevance for the issue of parental stress^(1,2,10)^ and motivated this study focused on pregnancy and the first month of childhood. Thus, the objective of the study was to identify factors related to parental stress in women during pregnancy and in the first month of the baby’s life.

## METHODS

### Design of Study

Prospective longitudinal study with descriptive and analytical data analysis.

### Local

The study was carried out in a medium-sized health district of a Brazilian municipality, with a Human Development Index (HDI) of 0.800, considered high in the country. It has a density of 1,080.47 inhabitants/km^2^, with 99.7% of its inhabitants living in the urban area^(11)^. The chosen health district has social characteristics that suggest a representative sample of the city’s population and has 12 health units with Family Health Strategy (FHS), considering that the largest number of them are in this region.

### Sample and Selection Criteria

The sample consisted of women with normal-risk pregnancies, longitudinally monitored in units with FHS, following the premise of starting prenatal care between November 2017 and June 2018, a pre-established time limit for the selection of possible participants. For the selection of participants, the support of the staff from the units mentioned and consultations at the municipal Health Information System (*SIS*) through access to electronic medical records were considered. Altogether, 529 participants were identified. With regard to the inclusion criteria, pregnant women of usual risk, over 18 years of age, residing in the area covered by the health district in which data collection was carried out and who were in the third trimester of pregnancy were considered, so as to maintain the bond during the data collection stages. This resulted in 246 potential participants, of which 220 agreed to participate, being included in the first stage of the investigation. In the second stage, 99 participants were excluded, due to: a) not being found for the second meeting after three attempts at telephone contact and a home visit, with 56 participants being considered as losses; b) change in the coverage area of the health district, considering 43 participants as a discontinuity criterion. A total of 121 participants composed the sample. This process can be seen in Figure S1 (Supplementary Material).

### Data Collection

After consideration and approval by the Research Ethics Committee, data collection was carried out between November 2018 and June 2019 by two professionals, one of whom was a nurse, both previously trained. Participants were contacted by telephone or at their homes, informed about the research and, after agreeing to participate, signed the Free and Informed Consent Form (FICF). The participants were interviewed in two moments, the first one during pregnancy and the second right after the baby completed the first month of life, during home visits. Validated questionnaires and scales were applied and a tablet with insertion of data on the platform *Fulcrum* ^®^ was used. Furthermore, the checklist for observational studies known as *STrengthening the Reporting of OBservational studies in Epidemiology* (STROBE)^(12)^ was used during the study.

### Study Variables

In the first stage, an interview was carried out and the genogram and ecomap were drawn up to identify the family structure and support network^(13)^. The independent variables characterizing the participants were: age, education, paid work, number of planned pregnancies, number of children, number of prenatal consultations, having a partner or not and whether he was the child’s father, living with a partner, use of alcoholic beverages, family income, receiving or not receiving government assistance, physical or psychological violence suffered and/or witnessed, feeling safe or not in the neighborhood, and the level of stress during pregnancy measured by the scale *Prenatal Psychosocial Profile* ^(14)^, translated and validated for Brazil^(15)^. Other independent variables such as number of people living in the same household and people living nearby were measured. Regarding family support networks, variables related to strong, weak, conflicting or non-existent bonds with father, mother, siblings, brothers/sisters-in-law, father-in-law, mother-in-law, friends and other relatives of the participating women were generated.

The interviewers received training in the standardization of genogram construction to avoid bias. The framework used was the Calgary family intervention and evaluation model^(16)^. The variables were coded for each participant and transcribed into the database. The scale *Prenatal Psychosocial Profile* used to assess stress during pregnancy is made up of 11 sentences, with answers about how much each of the situations explained causes stress to the pregnant woman. The accumulation of elements that make up these issues, such as the role of motherhood, satisfaction and maternal burden, for example, are listed as generators of stress during pregnancy.

In the second interview, carried out when the baby was one month old, the variables analyzed were: participant on maternity leave, receipt of financial resources, baby’s sex, type of delivery, delivery complications (or not), type of food provided to the baby, if the participant considers the child’s sleep a problem, and parental stress.

The study’s dependent variable was the level of parental stress. The Parental Stress Scale (PSSa) was applied^(17)^, validated for Brazilian Portuguese^(18)^, to measure the level of stress experienced by mothers/fathers of young children, specifically that produced by the parental role^(17)^. This scale is considered an instrument of easy application and free access, consisting of 16 items, distributed in four domains, based on a Likert scale scored as 0 = Strongly Disagree, 1 = Disagree, 2 = Undecided, 3 = Agree, and 4 = Strongly Agree. The PSSa structural model has two factors, with factor 1 called “parental satisfaction”, consisting of 8 items (1, 3, 4, 5, 6, 11, 15 and 16), and factor 2, “Parental stressors”, with 8 more items (2, 7, 8, 9, 10, 12, 13 and 14), which are inversely summed on the Likert scale. The calculation of results occurs from the sum of the scores of all items (Min. = 0; Max. = 64 points) and, the higher the score, the greater the parental stress. The average obtained by the sample shall be the parameter for stratifying the level of parental stress into low parental stress (equal to/below average) and high parental stress (above average)^(18)^.

### Data Analysis and Treatment

Data were exported from the platform *Fulcrum^®^*, with their tabulation and statistical calculations performed through the software IBM SPSS^®^ *Statistics* version 25 and R i386 version 3.4.0. For the data descriptive statistical analysis for each stage, the variables absolute frequency was calculated. Following the descriptive analysis, data were checked so that any discrepancies could be identified and corrected.

For quantitative variables, responses were stratified to calculate absolute frequency. The scales were analyzed according to the indication described by the respective authors.

The dependent variable ‘parental stress’ did not follow a normal distribution after performing the Kolmogorov-Smirnov test. To relate the categorical independent variables with the Parental Stress Level, Fisher’s exact test was performed. In the case of numeric variables, the Spearman correlation coefficient was used, with a 95% confidence interval. In associations, missings were not considered. The results of the association and correlation tests were considered statistically significant when the probability of rejecting the null hypothesis was less than 5% (*p*≤ 0.05).

To identify the relationship between the studied variables and parental stress (score and stress level), we used multivariate linear and logistic regression models in the *Rstudio* (version 2021.09.2), using native libraries of *R* (version 3.63). Linear regression was performed with the function *lm* (linear model) and logistics with the function *glm* (generalized linear model). The figures were generated with the package *sjPlot* (https://strengejacke.github.io/sjPlot/).

### Ethical Aspects

The study was part of a larger project approved by the Ethics Committee under opinion no. 2.239.180 in 2017 and amendment substantiated under opinion no. 3.550.644 in 2019 for the inclusion of other researchers. Each participant was provided with information about the objectives and performance of the study. All participants signed a two-way FICF, signed by the researcher in charge. One copy was given to the participants and the other was retained by the researcher. The guidelines that regulate research involving human beings, approved by Resolution CNS 466/2012, were followed.

## RESULTS

Among the 121 pregnant women, most (89.2%) were between 18 and 35 years old, with 11 to 13 years of study (50.4%), self-declared white (47.9%), and worked at home (52.9%) (Table S1 Supplementary Material). They had a partner who was the child’s father (90.9%) and lived in the same house as him (86.8%), planned one or more pregnancies (57.8%), it was not the first pregnancy (62, 8%), and the expected number of prenatal consultations (92.6%) were performed. Regarding monthly income, most participants (42.2%) earned between 1 and 2.5 minimum wages (R$ 998.00 in 2019) and about 15.7% had government financial assistance.

In the interview at the time of pregnancy, the participant was asked if she had suffered or witnessed any physical or psychological violence in the last three months. A portion of 13.2% responded that they suffered and 19.8% witnessed physical and/or psychological violence in the period. About 5% of pregnant women consumed alcoholic beverages during pregnancy and 29.8% considered their home neighborhood safe.

The construction of the genogram and ecomap identified the characteristics of the participants’ families. The number of people living in the same house ranged from 1 to 10, in addition to the participant herself and without considering the child at the time of pregnancy. Among the participants, 13.6% lived with a partner only, while 50.0% lived with a partner and already born child(ren) (Table S2 Supplementary Material). Other family configurations consisted of other people, such as siblings, mothers-in-law, fathers-in-law, brothers/sisters-in-law, cousins, uncles, stepmothers, stepfathers, and stepchildren. Participants also mentioned people who lived close to their homes, such as parents, mother/father-in-law, brother/sister-in-law, siblings, or other relatives.

As for family support networks, 23.1% of participants did not mention any support network. The others (76.9%) mentioned one or more support networks. There were 54 participants (44.6%) who mentioned FHS and 52 (42.9%) religious groups (Catholic, Evangelical, Spiritist, prayer and pastoral groups).

The notes on the bonds established by the women (Table S3 Supplementary Material) were 81.8% with one or more strong bonds with family members. The mother herself was the family member that the women most cited as a strong bond. Few participants mentioned having weak and conflicting bonds. However, the highest scores were for the parents-in-law as a weak bond and the mother-in-law as a conflicting bond.

As for stress during pregnancy, 67.8% had some stress and 32.2% had no stress during this period. Table 1 presents the relative frequency of the responses obtained from the participants in each item of the Perceived Stress Scale during pregnancy, used in the first stage. The question asked was ‘How much does each sentence cause you stress or concern at the moment?’, to which the participants chose a response between ‘no stress’, ‘some stress’, ‘moderate stress’, and ‘intense stress’.

**Table 1.** Frequency of participants’ answers (n = 121) of each item of the Perceived Stress Scale during pregnancy – Ribeirão Preto, SP, Brazil, 2019.

Item		Frequency (%)				
		No stress	Some stress	Moderate stress	Severe stress
1	Financial concerns	23.1	18.2	23.1	35.6
2	Feeling overwhelmed	38.0	14.9	16.5	30.6
3	Problems with the family	35.5	19.8	18.2	26.5
4	Recent loss of someone special	62.8	11.6	7.4	18.2
5	Current pregnancy	62.8	8.3	14.0	14.9
6	Problems at work	75.3	4.1	7.4	13.2
7	Having to change address	74.4	7.4	6.6	11.6
8	Be experiencing sexual, emotional, or physical violence/abuse	88.4	0.8	2.5	8.3
9	Problems with alcohol/drugs	92.6	2.5	0.8	4.1
10	Problems with friends	94.2	3.3	1.7	0.8

The issues with the highest scores as intense stress were financial concerns and feelings of overload. When the scores for moderate stress and severe stress are added, aspects of financial concerns, feelings of burden and problems with the family also stand out. The aspects pointed out with no stress, for most of the participants, were problems related to friends, alcohol and drugs, and suffering some type of violence.

In the second stage, when the baby had completed one month of life, the information was that 32.2% participants were on maternity leave and 24.0% were not, as well as 43.8% did not answer this question, for not working outside and not being entitled to a leave.

The babies’ profile showed the following characteristics: most were male (55.4%), vaginal delivery (62.0%), and with some complications during delivery (26.4%). With regard to breastfeeding, 67.8% were exclusively breastfeeding, 18.9% on predominant breastfeeding, 8.4% reported practicing mixed breastfeeding, and 4.9% were weaned in the first month of life. Regarding the baby’s sleep during this period, 13.2% of participants considered children’s sleep habits a problem in the first month after the child’s birth.

Regarding the imbalance in the maternal role, there is a slight oscillation between those with a high level of parental stress (47.9%) and low level of parental stress (52.1%). The Parental Stress Scale used in the child’s first month had agreement responses in 16 items. Table 2 describes the items and their frequencies. Items 1, 3, 4, 5, 6, 15 and 16 are positive sentences about parenting and disagreement with the presented sentence is related to greater parental stress. The other items are sentences that, with greater agreement, indicate higher levels of parental stress.

**Table 2.** Frequency of participants’ responses (n = 121) of each item of the Parental Stress Scale – Ribeirão Preto, SP, Brazil, 2019.

Item		Frequency (%)					
		Strongly disagree	Disagree	Undecided	Agree	Strongly agree
1	I am happy in my role as a parent.	0	0	0	28.1	71.9
2	Caring for my child sometimes takes more time and energy than I have to give.	17.4	15.7	3.3	42.1	21.5
3	I feel close to my child	0	0	0	28.1	71.9
4	I enjoy spending time with my child.	0	0	0	28.1	71.9
5	My child is an important source of affection for me.	0	0	0	24.8	75.2
6	Having children gives me a more optimistic view of the future.	0	1.7	4.9	34.7	58.7
7	The major source of stress in my life is my child.	69.5	23.1	3.3	4.1	0
8	Having children leaves little time and flexibility in my life.	20.7	29.7	4.9	36.4	8.3
9	Having children has been a financial burden.	42.1	38.0	8.3	9.9	1.7
10	It is difficult to balance different responsibilities because of my child.	21.5	30.6	9.0	30.6	8.3
11	My child’s behavior is often embarrassing or stressful for me.	57.8	36.4	1.7	3.3	0.8
12	If I had it to do over again, I might decide not to have children.	48.9	38.0	3.3	4.9	4.9
13	I feel overwhelmed by the responsibility of being a parent.	35.6	28.9	7.4	22.3	5.8
14	Having children has meant having too few choices and little control over my life.	40.6	38.0	4.9	11.6	4.9
15	I am satisfied as a parent	0	0.8	1.7	29.7	67.8
16	I find my children enjoyable.	0	0	0	25.6	74.4

The answers to item 2 ‘Taking care of my child sometimes takes more time and energy than I have to give’ and item 8 ‘Having children leaves little time and flexibility in my life’ were highlighted, due to the expressive number of participants signaling agreement. In addition to them, it is also possible to point out the relevance of the responses to items 10 ‘It is difficult to balance different responsibilities because of my child’, 13 ‘I feel overwhelmed by the responsibility of being a parent’, and 14 ‘Having children has meant having too few choices and little control over my life’, given that the participants’ agreement with these items also shows the high level of parental stress found.

The associations between individual, family and social characteristics, and the frequency of parental stress in women, made using Fisher’s exact test, are described in Table 3. According to the analysis, none of the characteristics studied showed statistically significant difference and, therefore, showed no association with high levels of parental stress (values of *p*> 0.05).

**Table 3.** Distribution of individual, family and social characteristics and the frequency of parental stress in women (n = 121) – Ribeirão Preto, SP, Brazil, 2019.

Variables		Frequency n (%)		Value of *p* ^a^
		Low parental stress	High parental stress	
Age (years)	18–25	48.1	51.9	0.730
	26–35	55.6	44.4	
	≥ 36	23.1	76.9	
Education (years)	≤ 7	64.7	35.3	0.358
	8 to 10	42.9	57.1	
	11 to 13	50.8	49.2	
	≥ 14	71.4	28.6	
	Missing: 1	–	–	
Paid work	Yes	54.4	45.6	0.716
	No	50.0	50.0	
Has a partner	Yes	51.8	48.2	1.000
	No	55.6	44.4	
Children (No.)	0	44.4	55.6	0.137
	1–2	59.7	40.3	
	≥ 3	33.3	66.7	
Receives government assistance	Yes	31.6	68.4	0.078
	No	56.4	43.6	
	Missing: 1	–	–	
Has suffered physical or psychological violence in the last three months	Yes	37.5	62.5	0.284
	No	54.3	45.7	
Has witnessed physical or psychological violence in the last three months	Yes	50.0	50.0	0.824
	No	52.6	47.4	
Community safety	Yes	52.9	47.1	0.843
	No	50.0	50.0	
Intake of alcoholic beverage	Yes	50.0	50.0	1.000
	No	52.2	47.8	
Maternity leave	Yes	46.2	53.8	0.438
	No	54.9	45.1	
Has the right for a leave	Yes	48.6	51.4	1.000
	No	33.3	66.7	
	Missing: 82	–	–	
Type of delivery	Vaginal	53.3	46.7	0.852
	Cesarean section	50.0	50.0	
Complications in childbirth	Yes	50.0	50.0	0.838
	No	52.8	47.2	
Exclusive breastfeeding	Yes	51.2	48.8	0.847
	No	53.8	46.2	
Considers the child’s sleep a problem	Yes	43.8	56.3	0.594
	No	53.3	46.7	

^a^ Value of *p* obtained from the analysis with Fisher’s exact test.

In the correlation analysis between numerical variables and the total parental stress score (Table 4), the test showed significant results for the number of ‘planned pregnancies’ and for ‘stress during pregnancy’ (*p*< 0.05). These findings indicate a correlation between these factors and parental stress for the women in the present study.

**Table 4.** Spearman’s correlation coefficient between numerical independent variables and total parental stress score in women (n = 121) – Ribeirão Preto, SP, Brazil, 2019.

Variables	N^a^	r^b^	Confidence interval of 95%	Value of *p*
Planned pregnancies (No.)	119	–0.237	[–0.433 –0.040]	**0.009**
Family income (BRL)	114	0.000	[–0.187 0.188]	1.000
Residents in the same house (no.)	118	0.087	[–0.125 0.298]	0.350
Support networks (No.)	121	0.076	[–0.110 0.263]	0.405
Strong bonds (No.)	121	–0.011	[–0.175 0.153]	0.907
Weak bonds (no.)	121	–0.136	[–0.270 –0.0007]	0.138
Conflicting bonds (No.)	121	0.143	[–0.014 0.300]	0.117
Stress during pregnancy (score)	121	0.194	[0.030 0.418]	**0.033**

^a^ Number of participants who answered this question. ^b^Spearman coefficient.

**Figure 1.**
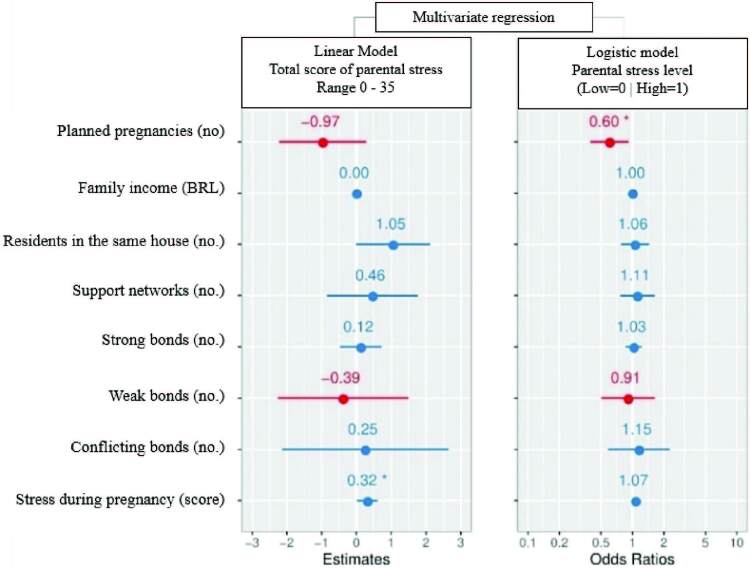
Forest plot obtained from linear (left) and logistic (right) multivariate regression models using as dependent variables, respectively, the total score of parental stress and level of parental stress and the characteristics of the participants as independent. The points represent the estimates for linear regression or *odds ratio* for logistic regression and the bars indicate the 95% confidence interval. Positive estimated values or *odds ratio* greater than units are represented in blue and negative or below unity are represented in red. Calculations were performed on a data set of 110 participants out of a total of 121 (11 missing). *p< 0.05.

Analyses with multivariate regression models were performed to identify possible relationships between the total parental stress score or the level of parental stress and the individual, family and social characteristics investigated (Figure 1). Considering the total parental stress score, a significant difference was identified for the factor ‘stress during pregnancy’, indicating a relationship between this variable and the highest total scores of parental stress (positive *Odds Ratio*). Thus, for every one point increase in the pregnancy stress score, it is estimated that the total parental stress score increases by 0.32.

Considering the level of parental stress categorically (high stress = 1; low stress = 0), the ‘number of planned pregnancies’ was the only variable that showed statistical difference (*Negative Odds Ratio*). With this result, it is estimated that, for each increase of one point in the number of planned pregnancies, the level of parental stress decreases by 0.40.

## DISCUSSION

In the present study, it was identified that stress during pregnancy is directly related to total parental stress scores. The presence of stress during pregnancy and high parental stress for most participants corroborates the scientific literature. Having some parental stress is expected and considered within the normal range, but high levels of stress for parenting can affect the relationship between parental caregivers and children^(1,2)^.

The major concern is that parental stress patterns may negatively affect parenting practices and early childhood development^(18)^. Studies point out that daily stress, the relationship that parental figures had with their own fathers/mothers and the presence of psychopathologies suggest enhancement of parental stress^(1–3,19)^.

Motherhood for women is related to the first interactions and identification as a woman, during childhood, adolescence and pregnancy, with pregnancy being a significant process that involves feelings of joy, sadness, satisfaction, and dissatisfaction^(8)^. The moment of pregnancy, in some way, requires dealing with and assuming the maternal role, generating changes in their relationships and lifestyle, in the psyche and in the woman’s body^(20)^. Most pregnant women who participated in the present investigation showed some stress during the gestational period. In addition, stress during pregnancy was correlated with high parental stress. It is essential to embrace the women’s ambiguous feelings, listen to their concerns and doubts and work on empowerment, so that they feel able to exercise motherhood and strengthen their existence as a person and a woman, resulting in circumstances to reduce parental stress.

In this investigation, the results showed that it can be estimated that the number of planned pregnancies decreases the level of parental stress. Not planning a pregnancy is a stressful process, given that decision-making and preparation for a pregnancy are related to lesser amounts of stress for parental caregivers^(2,3)^. Reproductive health is a necessary factor to be worked on in consultations, to allow pregnancy planning, aiming at building adequate parenting that promotes less parental stress and creates better environments for the development of children and women.

The family designates several realities that can generate complex circumstances and with many meanings that vary according to each person’s perspective^(21)^. The structure and family dynamics can permanently influence the child’s life, affecting their health and well-being^(3,5,19)^. However, other aspects of the family were not associated with parental stress in the baby’s first month of life, such as interpersonal bonds and the presence of support networks in this study.

Violent environments, lack of financial and material resources, unemployment, food insecurity, intrafamily violence, negligence, abuse, criminality, parental psychopathology, alcoholism, and use of illicit drugs, with losses and abandonment, pose multiple risks to children development^(22)^. Therefore, such aspects deserve continuous investigations to analyze different circumstances and their repercussions for the health of women and children.

Stressful experiences in the labor and birth process can generate stress^(17,23)^ and impact parenting interactions with the baby^(8,24)^. Despite this, in this study the other factors analyzed did not show results related to parental stress. Parenting encompasses a complex context of relationships that, depending on the vulnerabilities, has the potential to generate stress to the individuals involved, especially with regard to the baby’s first month of life, since it is a period marked by birth, arrival at home, and by the initial processes of care and adaptation by the mother and family, which in itself can justify the presence of parental stress^(25,26)^.

The use of the genogram and ecomap brought potentialities and challenges to the present study. Such tools contributed to establish interaction, describe families, and identify variability in family arrangements, interpersonal bonds, and elements of the support network. The genogram and the ecomap were therefore configured as facilitators in the identification of stressful conditions. This way, they reaffirm their relevance to clarify the internal and external structures of families, providing data collection and ways of approximation between interviewer and interviewee^(13)^.

Effective practices for promoting the child’s development involve observation, detection and intervention, and it is extremely important to know family relationships and parental stress processes. In this regard, it is necessary to address daily care with families, integrated with intersectoral actions, with emphasis on health, education, and social protection^(4,27)^.

Parenting practices are a broad universe, determined by a cultural, social, and emotional environment^(3,22)^. It is possible to help parental caregivers by identifying strengths, capabilities, and resources that reinforce positive parenting practices and styles, to directly impact child development^(20,28)^. At the same time, the increase in public policies aimed at very early childhood, from the prenatal period to the first three years of age of children, is critical for solid human development^(13,29)^.

The limitations of the present study refer to the identification of parental stress centered on interviews with women, and it is worth expanding to different parental caregivers and at different moments of pregnancy and childcare. Furthermore, it should be noted that losses and discontinuity of sample participants is considered a limiting factor for the study. The object of study outlined here is complex and broad and other studies are relevant in the theme of parenting practices, support networks, and adversities in very early childhood.

## CONCLUSIONS

Women who had a higher level of parental stress had stress during pregnancy, and pregnancy planning proved to be a factor in reducing the level of stress. These results suggest that actions to early identify and reduce parental stress are essential, with emphasis on the work of health professionals with families in the context of primary health care.

In addition to concerns about children’s survival, providing qualified support to parental caregivers and families brings benefits to child development and to the well-being of children and women. This way, the increase of good practices in the prenatal period and in the first years of children’s lives is essential for comprehensive health with far-reaching repercussions.

## SUPPLEMENTARY MATERIAL

The supplementary material is available in: https://data.scielo.org/dataset.xhtml?persistentId=doi:10.48331/scielodata.TB4JUB&version=1.0
